# Early Antiretroviral Therapy Is Associated with Lower HIV DNA Molecular Diversity and Lower Inflammation in Cerebrospinal Fluid but Does Not Prevent the Establishment of Compartmentalized HIV DNA Populations

**DOI:** 10.1371/journal.ppat.1006112

**Published:** 2017-01-03

**Authors:** Michelli F. Oliveira, Antoine Chaillon, Masato Nakazawa, Milenka Vargas, Scott L. Letendre, Matthew C. Strain, Ronald J. Ellis, Sheldon Morris, Susan J. Little, Davey M. Smith, Sara Gianella

**Affiliations:** 1 Department of Medicine, University of California San Diego, La Jolla, California, United States of America; 2 HIV Neurobehavioral Research Center, San Diego, California, United States of America; 3 Departments of Neurosciences and Psychiatry, University of California San Diego, La Jolla, California, United States of America; 4 Veterans Affairs San Diego Healthcare System, San Diego, California, United States of America; Vaccine Research Center, UNITED STATES

## Abstract

Even when antiretroviral therapy (ART) is started early after infection, HIV DNA might persist in the central nervous system (CNS), possibly contributing to inflammation, brain damage and neurocognitive impairment. Paired blood and cerebrospinal fluid (CSF) were collected from 16 HIV-infected individuals on suppressive ART: 9 participants started ART <4 months of the estimated date of infection (EDI) (“early ART”), and 7 participants started ART >14 months after EDI (“late ART”). For each participant, neurocognitive functioning was measured by Global Deficit Score (GDS). HIV DNA levels were measured in peripheral blood mononuclear cells (PBMCs) and CSF cell pellets by droplet digital (dd)PCR. Soluble markers of inflammation (sCD163, IL-6, MCP-1, TNF-α) and neuronal damage (neurofilament light [NFL]) were measured in blood and CSF supernatant by immunoassays. HIV-1 partial *C2V3 env* deep sequencing data (Roche 454) were obtained for 8 paired PBMC and CSF specimens and used for phylogenetic and compartmentalization analysis. Median exposure to ART at the time of sampling was 2.6 years (IQR: 2.2–3.7) and did not differ between groups. We observed that early ART was significantly associated with lower molecular diversity of HIV DNA in CSF (p<0.05), and lower IL-6 levels in CSF (p = 0.02), but no difference for GDS, NFL, or HIV DNA detectability compared to late ART. Compartmentalization of HIV DNA populations between CSF and blood was detected in 6 out of 8 participants with available paired HIV DNA sequences (2 from early and 4 from late ART group). Phylogenetic analysis confirmed the presence of monophyletic HIV DNA populations within the CSF in 7 participants, and the same population was repeatedly sampled over a 5 months period in one participant with longitudinal sampling. Such compartmentalized provirus in the CNS needs to be considered for the design of future eradication strategies and might contribute to the neuropathogenesis of HIV.

## Author Summary

Human Immunodeficiency virus (HIV) enters the central nervous system (CNS) early after infection and provides the basis for the development of neurocognitive impairment and potentially the establishment of latent reservoirs. Early initiation of antiretroviral therapy reduces HIV reservoir size in the periphery, but no previous study has assessed whether this strategy can also affect the HIV reservoir in the CNS. In this study, we prospectively collected and evaluated cerebrospinal fluid (CSF) and peripheral mononuclear blood cells (PBMC) from a cohort of 16 HIV-infected participants on suppressive antiretroviral therapy (ART) who started ART early (<4 months) and late (>14 months) after the timing of HIV infection. We found that early ART initiation was associated with lower molecular diversity of HIV DNA and lower levels of inflammatory markers in CSF in comparison to late ART start. We also found evidence of compartmentalized HIV DNA populations between the CSF and blood in the majority (75%) of the participants with available paired sequences, including two (66%) participants from the early ART group. Such compartmentalized provirus in the CNS will be important for the design of future eradication strategies and could contribute to the neuropathogenesis of HIV.

## Introduction

Human Immunodeficiency Virus (HIV) invades the central nervous system (CNS) early during the course of infection [1,2] providing the foundations for neurocognitive impairment (NCI) and potentially establishing a latent reservoir [3,4]. Newly infected individuals typically have homogeneous HIV populations in blood [5,6] that evolve during untreated infection to generate diverse viral variants [2,7,8]. Compartment-specific selective pressures can subsequently lead to the emergence of unique HIV populations in different anatomical sites during the course of infection, including the CNS [2,7,9–11], the genital tract [12], and other tissues [13,14]. HIV RNA variants can be sequestered from blood into the CNS early after infection (within 2–6 months) and give rise to a separate HIV RNA population in the cerebrospinal fluid (CSF) [2,8], which remains genetically distinct from blood throughout the course of infection. Overall, these observations suggest that the CNS can be permissive for HIV replication from a very early period after HIV infection. The presence of compartmentalized HIV variants within the CNS has important implications: (1) compartmentalization of HIV RNA in CNS has been associated with greater inflammation and worse neurocognitive outcomes [15–17] and, (2) independent replication of HIV within the CNS might hinder HIV eradication efforts by providing a distinct reservoir of HIV persistence different from that found in peripheral CD4^+^ T cells. This has been suggested by previous observations reporting differential emergence of drug resistance mutations between CSF and blood during antiretroviral therapy (ART) failure [18–20].

Combination ART has markedly reduced the incidence of HIV-associated dementia [21,22]. However, the true impact of early ART initiation on HIV-associated neurocognitive impairment is still under investigation [23]. While the viral replication and evolution of HIV RNA in the CNS has been extensively studied even during early HIV infection [2,8,24,25], little is known about the HIV DNA populations persisting in this anatomic compartment during the earliest phase of HIV infection, and especially during suppressive ART. Similar to blood [26,27], initiation of ART during early HIV infection might limit the diversification of HIV DNA within the CNS, affecting the size and molecular diversity of the HIV reservoir, preventing inflammation, and limiting brain damage. But these features have not been evaluated yet for the CNS. Our study used a unique set of samples from a well-characterized cohort of HIV-infected individuals followed longitudinally from early HIV infection to investigate the effects of early ART initiation on the size and molecular and phylogenetic characteristics of the HIV DNA populations while on long-term suppressive ART. Additionally, since chronic inflammation has been associated with HIV persistence [28], we evaluated the effects of early ART on selected inflammatory markers in blood and CSF supernatant.

## Results

### Study population and samples

Study participants (n = 16) were all HIV-infected males with a median age of 41 years (Inter Quartile Range [IQR]: 32.5–52.5) selected among participants of the San Diego Primary Infection Resource Consortium (SD PIRC). At baseline (pre ART), the median plasma viral load was 176,000 HIV RNA copies/μl (IQR: 40,287–515,900). Participants achieved viral suppression after a median of 76 days (IQR: 47–256) ART start and remained undetectable during the entire follow-up (median of 3.5 viral load measurements per participant, median of 168 days between visits, median % of time-points with suppressed HIV RNA during follow-up 100%). Participants received ART for a median duration of 2.6 years (IQR: 2.2–3.7) and had suppressed levels of HIV RNA in blood plasma (<50 copies/ml) and in the CSF supernatant (at single copy level) at the time of sample collection. Six out of sixteen participants were on a protease inhibitor (PI)-based ART regimen, 6/16 were on a non-nucleoside reverse transcriptase inhibitor (NNRTI)-based regimen and 4/16 on an Integrase Strand Transfer Inhibitor (INSTI)-based regimen, all in combination with two nucleoside reverse transcriptase inhibitors (NRTI).

While we recruited participants with early and late ART initiation according to study design, the exact categorization (<4 months or >14 months) was performed retrospectively to participant enrollment, but a priori to any molecular data generation or interpretation. The “early ART group” (n = 9) started ART within a median of 1.8 months from estimated date of infection (EDI) (IQR: 1.5–3) while the “late ART group” (n = 7) started ART within a median of 17.2 months from EDI (IQR: 14.8–30.9). Detailed demographic and clinical characteristics of the study population are summarized in Table 1. No significant differences between the early and late ART groups were observed for any recorded demographic or clinical characteristics (p>0.2). Paired CSF and blood samples were obtained at baseline from all 16 participants. Two participants (both belonging to the late ART group) agreed to donate CSF and blood at a second (T0338 and T0366) and a third (T0366) longitudinal visit. These additional samples were obtained 5 and 3 months from the first evaluation and 2 months from the second evaluation, respectively.

**Table 1:** Baseline demographic and clinical characteristics, HIV DNA and inflammatory markers levels between different ART initiation groups.

Parameters	All participants (n = 16)	Early ART (N = 9)	Late ART (N = 7)	p-value (Mann-Whitney)
**Characteristics**				
Age^a^	41 [32.5, 55]	44 [40, 57]	35 [29.5, 47.5]	0.22
Education (years) ^a^	14 [12,16]	13.5 [12, 16]	15 [13.5, 16.5]	0.60
Male, %	100	100	100	1.00
Caucasian, %	86.6	77.8	100	0.51
Current CD4^a^	726 [603, 1051]	786 [583, 1292]	726 [603, 816]	0.59
Nadir CD4^a^	374 [287, 505]	473 [300, 592]	373 [300, 414]	0.52
Estimated Duration of infection (years) ^a^	3.4 [2.3, 5.1]	2.7 [2.1, 3.4]	5.1 [4.4, 5.7]	
Exposure LT ARVs (years)^a^	2.6 [2.2, 3.7]	2.8 [2.1, 3.7]	2.6 [2.4, 3.2]	0.81
CNS Penetration Effectiveness^a^	7 [4, 7]	6.5 [4, 7]	7 [4, 7]	0.93
GDS^a^	0.2 [0.1, 0.9]	0.6 [0.1, 1.1]	0.2 [0.1, 0.2]	0.38
**CSF**				
HIV DNA (cps/million cells)^b^	2,701.5 [1118.9, 4526.2]	10,559.7 (N/A^c^)	1,554 [578.9, 3078.7]	0.34
Diversity (%)	1.1	0.9	2.5	0.11
Cytokines^a^				
sCD163 (ng/mL)	45.8 [37.5, 63]	39.7 [35.6, 62.6]	49.4 [40.4, 63.2]	0.60
IL-6 (pg/mL)	1.0 [0.9, 1.2]	0.9 [0.6, 1.1]	1.2 [0.9, 1.4]	**0.03**
MCP-1 (pg/mL)	376.9 [339, 437.5]	414.9 [343.8, 447.3]	344.8 [330, 377]	0.17
TNF-α (pg/mL)	0.24 [0.1, 0.3]	0.2 [0.02, 0.2]	0.3 [0.3, 0.5]	**0.02**
NFL (ng/L)	168.1 [109.7, 270.5]	173.8 [148.5, 269.8]	138.7 [109.2, 245.5]	0.53
**Blood**				
HIV DNA (cps/million cells)	18.6 [9, 44.2]	14.1 [10.1, 37]	39.1 [7.1, 62.7]	0.62
Diversity (%)	2.5	2.1	2.5	0.26
Cytokines^a^				
sCD163 (ng/mL)	648.6 [479.2, 826.4]	790.5 [560.9, 870.5]	508.2 [478.9, 570.1]	0.20
IL-6 (pg/mL)	0.5 [0.2, 0.7]	0.4 [0.2, 0.6]	0.7 [0.5, 0.7]	0.68
TNF-α (pg/mL)	1.7 [1.3, 2.3]	1.7 [1.3, 2.1]	1.8 [1.4, 3.2]	0.80
MCP-1 (pg/mL)	118.7 [103.5, 145.8]	126.1 [106, 149.1.]	113.9 [95.4, 131.9]	0.52

GDS = Global Deficit Score; ART = antiretroviral therapy; LT = lifetime.

^a^Data shown as median [interquartile range].

^b^Data shown as median [interquatile range] among HIV DNA detectable samples. Fisher test p value is shown.

^c^Not computed because only two values were available for this group.

### HIV DNA levels and detectability in CSF cells and PBMC

Overall, this study comprised 16 participants with baseline samples (9 early ART and 7 late ART) and 3 extra time points from 2 participants (both belonging to the late ART group). Among the 16 baseline samples, we detected HIV DNA from 6 CSF cell pellet samples (37.5%) by ddPCR and amplified the HIV partial *env* gene (C2V3, HXB2 coordinates 6,928–7,344) in 8 CSF cellular samples (50%) by nested PCR (Summarized in Supplementary S1 Table). For the purpose of our study, we considered as “positive” any CSF sample with detectable HIV DNA by either ddPCR or nested PCR (or both). This resulted in 10 HIV DNA positive CSF samples at baseline (62.5%, 5 in the early ART and 5 in the late ART group) and 6 undetectable (negative for both ddPCR and nested PCR). Of the 3 extra time point samples (longitudinal), we detected HIV DNA from one CSF cellular sample by ddPCR (T0338 TP2) but we were able to amplify C2V3 *env* in all 3 CSF cellular samples (T0338 TP2 and T0366 TP2 and TP3).

Of note, only 5 samples (out of the 13 with detectable HIV DNA) had consistent detection of HIV DNA by ddPCR and nested PCR across both aliquots. This inconsistency across aliquots is not surprising because of the low number of infected cells which increases the proportional impact of unequal cell numbers across the two separate aliquots during processing. Also, the dilution of lysates before the ddPCR droplet generation may have significantly reduced the sensitivity of the ddPCR assay.

When comparing the two groups, HIV DNA was detected in 5 out of 9 CSF cell pellet samples tested as part of the early ART group and in 5 of 7 in the late ART group, but this difference was not statistically significant (55% versus 71%, relative risk 0.78, p = 0.63); HIV DNA was detected in all but one (93.8%) of the 16 PBMC samples.

### HIV DNA molecular characteristics in blood and CSF cells

To further characterize the HIV DNA population, we sequenced partial *env* from CSF cell pellets (n = 8) and PBMCs (n = 14) at baseline. For two participants, we also obtained partial *env* sequences from one additional time-point (T0338 and T0366). Detailed characteristics of the viral sequences are provided in supplementary S2 Table (for PBMC) and S3 Table (for CSF cell pellets).

**Fig 1:**
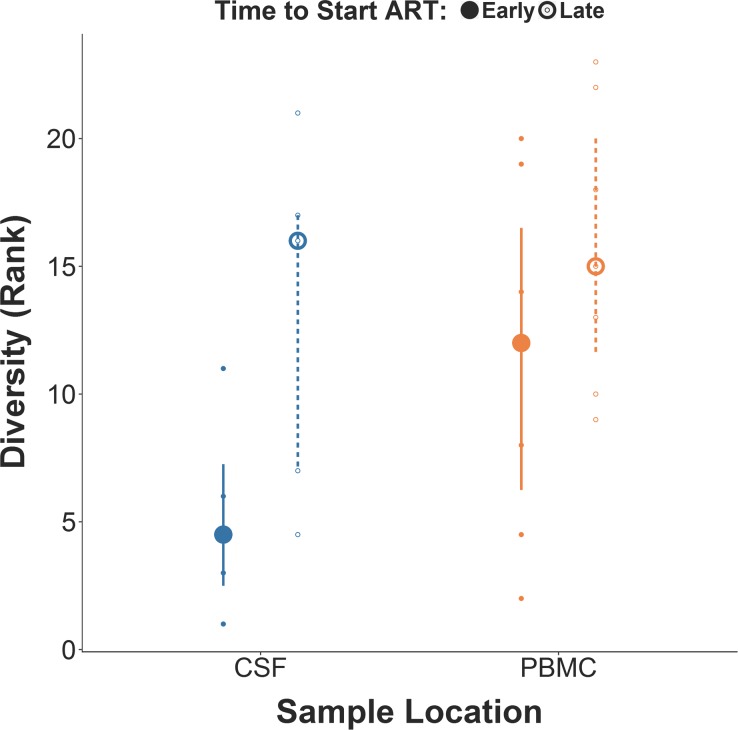
Comparison of molecular diversity for HIV DNA (partial *env* gene) in CSF cells and PBMC between early ART versus late ART groups. Mann Whitney comparison between early ART versus late ART groups for rank-transformed percentage of molecular diversity for HIV-1 *env* in CSF cells and PBMC. p values for both comparisons were>0.10.

Overall, participants in the early ART group presented a lower molecular diversity of the CSF HIV DNA population, as compared to the late ART group (Fig 1; Median: 0.9% versus 2.5%, p = 0.11). In contrast, no difference in molecular diversity was observed in the PBMC HIV DNA population between the two ART groups (Fig 1, Median: 2.1% versus 2.5%, p = 0.26). The CSF/PBMC diversity ratio was 0.58 (range: 0.31–0.69) for the early ART group and 0.84 (range: 0.33–1.06) late ART group (p = 0.12).

**Fig 2:**
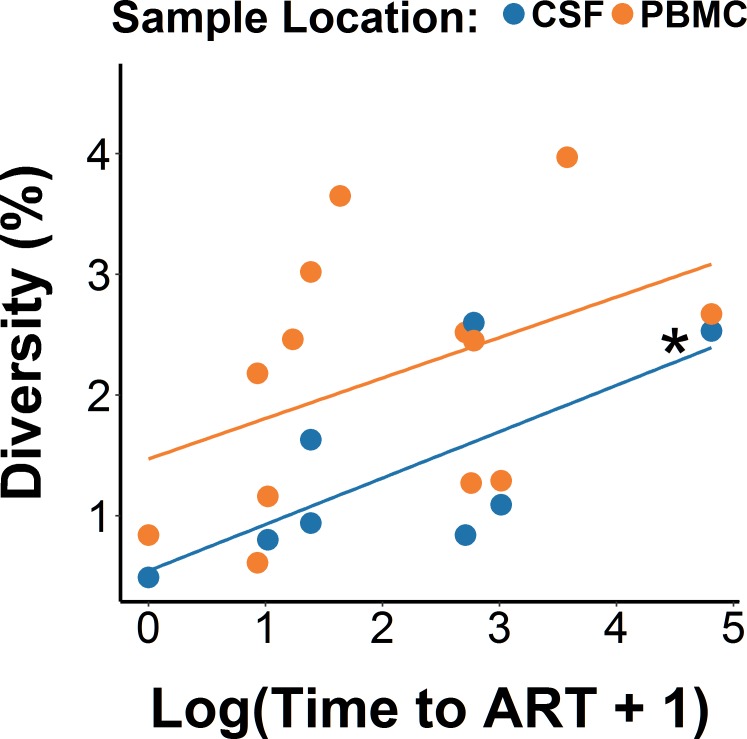
Regression analysis between time to ART start and HIV DNA molecular diversity in blood and CSF. Linear mixed-effects regression models revealing the relationship between molecular diversity of HIV DNA *env* with log-transformed time to ART start. *p = 0.05 for the correlation within CSF.

Next, we used a mixed-effects model where baseline viral diversity was predicted by log-transformed time to ART from EDI as a continuous variable to evaluate its association with percentage of diversity (Fig 2). We observed a higher percentage of diversity among participants with the longer time to ART from EDI, collapsed across blood and CSF (b = 0.36, p = 0.04, η^2^_p_ = 0.28). When evaluating the compartments separately, this association was significant in CSF (p = 0.05, η^2^_p_ = 0.22), but not in blood (p = 0.08, η^2^_p_ = 0.19). Diversity was significantly higher in PBMC than in CSF by 0.8% (p = 0.02, η^2^_p_ = 0.31), regardless of time to ART. We also included five covariates (age, peak viral load, CD4, CD8, and CD4/CD8 ratio) separately in the model to examine their potential effects on diversity and the association between time to ART and diversity. None of the covariates was significantly associated with diversity (all p-values>0.1, all η^2^_p_<0.05) while the association between time to ART start and diversity remained consistently significant (p-values<0.05).

The average number of input HIV DNA templates from CSF cells into the first round PCR reaction was estimated using the number of HIV DNA and RPP30 copies (based on our ddPCR data). The median HIV DNA copies per million cells among HIV-positive CSF cell samples was 2,701 copies/million cells (IQR: 1,119–4,526). The median number of CSF cells for each ddPCR reaction (estimated by RPP30) was 2,340 (IQR: 1120.5–2700 cells). After adjusting for the different volumes (5 μl for ddPCR and 10μl for nested PCR) and the dilution factor, we estimate that the average calculated HIV template input was 22 copies of HIV DNA (range: 4–64) per reaction. It should be noted that these levels are likely an under-estimate, given the inherent dilution with the ddPCR methods, as described in the method section and above.

To further evaluate if the low HIV DNA input for the sequencing reaction influenced our measures of molecular diversity, we performed additional sensitivity analyses based on our baseline model described above. We first assessed the potential impact of HIV DNA copies on diversity measures by including log-transformed HIV DNA levels (measured in blood and CSF when available) into our model; we found no statistical evidence that the number of HIV DNA copies was associated with any bias in molecular diversity (p = 0.21, η^2^_p_ = 0.10). Second, to take into account the lack of consistency across aliquots, we compared diversity measures between cases with consistent versus inconsistent detectability across aliquots (assuming that cases with ddPCR+/nested PCR+ will have higher HIV DNA levels compared to cases with ddPCR-/nested PCR+) and we did not find a significant difference (p = 0.46, η^2^_p_ = 0.04). While the ability to detect a significant effect in our sensitivity analysis was surely limited by the small sample size, this analysis suggests that the effect size of our primary predictor (time from EDI to ART, η^2^_p_ = 0.28) on molecular diversity of partial env was greater than the effect sizes of each covariate, including the number of template HIV DNA copies (η^2^_p_ = 0.10) and the number of positive aliquots (η^2^_p_ = 0.04). Finally, to test the consistency of the diversity measures across blood and CSF, we performed a correlation analysis, and found that molecular diversity in CSF pellets was significantly associated with molecular diversity in PBMC (Pearson r = 0.78, p = 0.02), strongly supporting the validity of our conclusion and measurements within the context of all the aforementioned limitations.

### Compartmentalization analysis of HIV DNA between CSF cells and PBMC

Paired HIV DNA sequences (partial *env*) from CSF cell pellets and PBMCs were obtained for 8 participants, 3 from the early ART group and 5 from the late ART group. Two individuals (both from the late ART group) had additional HIV DNA sequences from a second time-point available (obtained 3 and 5 months from the first evaluation, respectively). One individual had a third time-point (2 months apart). Compartmentalization was assessed using three distinct methods: distance-based F_ST_ test with and without collapsed haplotypes and tree-based Slatkin-Maddison (SM) test. Applying our conservative definition (i.e. significant compartmentalization for all three methods), we observed a significant genetic compartmentalization between the HIV DNA populations sampled from CSF cells and PBMCs in 6 of 8 participants, including 2 individuals in the early ART group (T0104 and T0430) (Table 2). Of note, the Fst estimates were congruent between both distance-based approaches, with and without collapsed haplotypes (Kendall τ test p<0.01).

**Table 2:** Compartmentalization Analysis for partial *env* HIV DNA in PBMC and CSF.

Subject	TP	ART Group	Location		Fst test*	p value	Slatkin Maddison p value	Compartmentalization
T0430		Early	PBMC	CSF	0.39	<0.01	0.03	Yes
T0073		Early	PBMC	CSF	-0.03	1	0.79	No
T0104		Early	PBMC	CSF	0.2	<0.01	<0.01	Yes
T0020		Late	PBMC	CSF	0.08	0.04	0.04	Yes
T0133		Late	PBMC	CSF	0.33#	<0.01	0.03	No
T0156		Late	PBMC	CSF	0.37	<0.01	<0.01	Yes
T0338	TP1	Late	PBMC	CSF	0.33	<0.01	<0.01	Yes
	TP2		PBMC	CSF	0.16	<0.01	0.01	Yes
T0366	TP2	Late	PBMC	CSF	0.24	<0.01	<0.01	Yes
	TP3		PBMC	CSF	0.21	0.05	0.03	Yes

Compartmentalization is called conservatively when all three tests indicate compartmentalization [see text].

#Indicates tests which become significant (or not significant) if copy numbers are ignored during F_ST_ calculations. [see text]. Statistical significance was derived via a 1,000-fold population-structure permutation test. TP: Time-point, CSF: Cerebrospinal Fluid; PBMC: Peripheral Blood Mononuclear Cells.

*While F_ST_ could assume negative values, none of the samples with negative F_ST_ could be called compartmentalized.

### Phylogenetic structure of the HIV DNA populations

**Fig 3:**
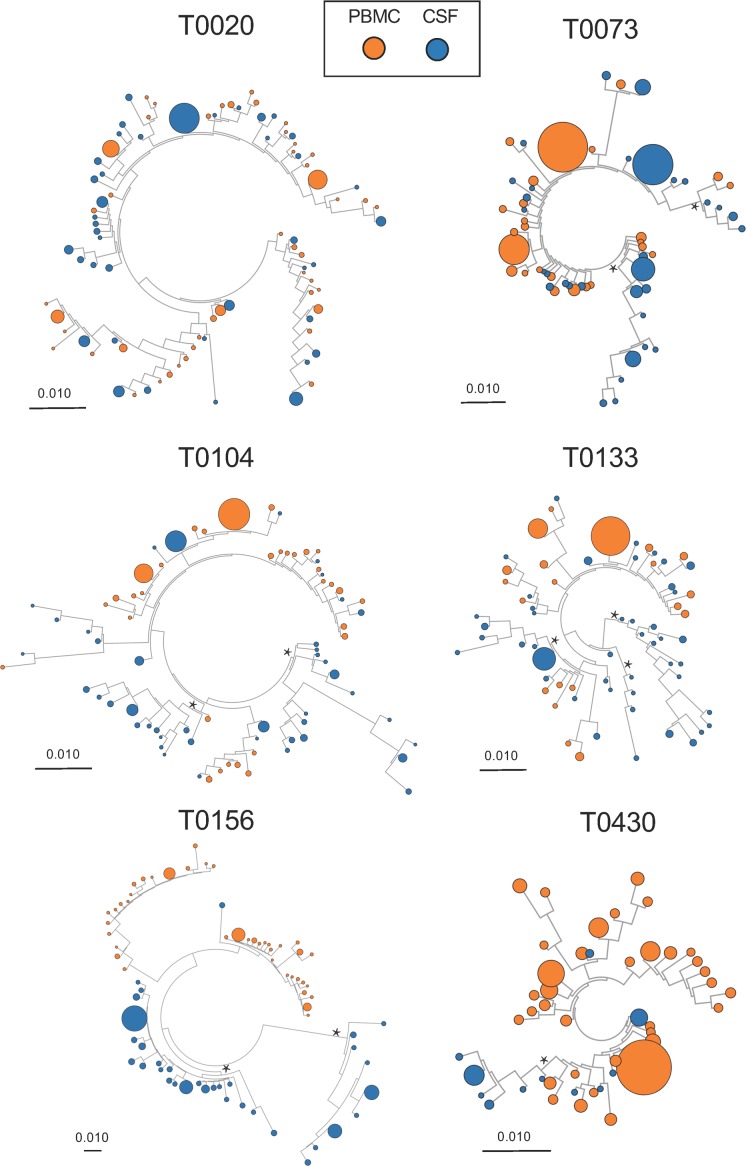
Maximum likelihood phylogenetic trees of partial HIV *env* sequences from paired CSF cellular pellets and PBMC sampled at baseline in 8 participants. HIV DNA populations were compartmentalized in the CSF for 6 baseline samples. Monophyletic HIV DNA populations in the CSF (i.e. aLTR>0.9, see asterisks) were found in 2 participants who initiated ART during late (T0133 and T0156) and 3 who initiated ART during early infection (T0104, T0073 and T0430). Genetic scale distances of 0.01 number of nucleotide substitutions per site.

**Fig 4:**
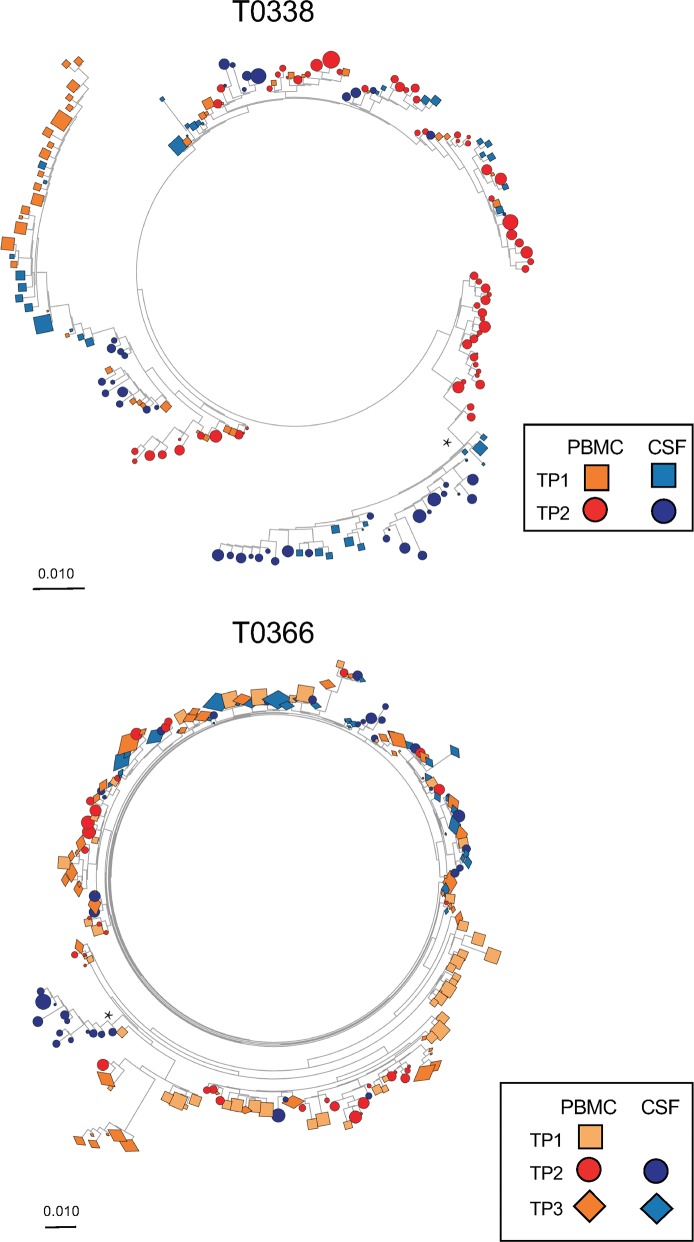
Maximum likelihood phylogenetic trees of partial HIV *env* sequences from paired CSF cellular pellets and PBMC sampled longitudinally in 2 participants. HIV DNA populations were compartmentalized in the CSF at baseline samples and persisted over time during suppressive ART. Monophyletic HIV DNA populations in the CSF were found in one participant (T0338) who initiated ART during late HIV infection and persisted overtime (see asterisk). Genetic scale distances of 0.01 number of nucleotide substitutions per site.

Maximum likelihood (ML) phylogenetic trees were created to evaluate the structure of the HIV DNA populations for participants with paired *env* sequences from CSF cells and PBMCs (Fig 3 and Fig 4).

Tree topologies revealed the presence of monophyletic HIV DNA populations in CSF for 7 participants (Figs 3 and 4, indicated with an asterisks). Two (T0104 and T0430) of the six individuals with evidence of well-segregated viral populations in the CSF were part of the early ART group. The same monophyletic CSF virus population was sampled from longitudinal CSF pellets over a period of 5 months for the one individual with a second time-point (T0338; Fig 4, see asterisk).

### Soluble markers of inflammation and neuronal damage

**Fig 5:**
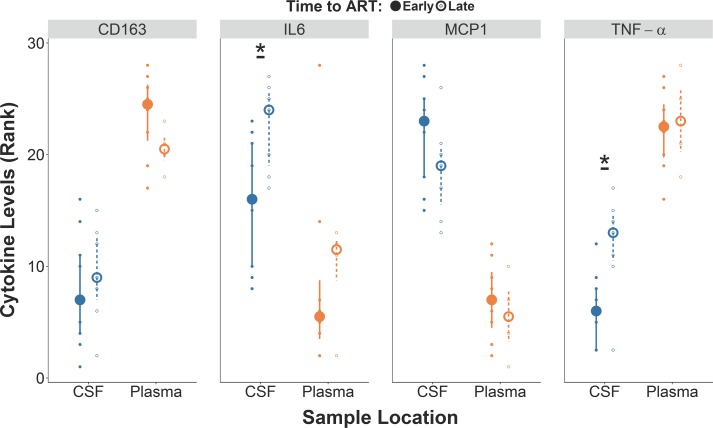
Levels of CSF inflammatory markers were lower in the early ART group in comparison to late ART group. Comparison between early ART versus late ART groups for rank-transformed levels of CD163, IL-6, MCP1, and TNF-α. *p<0.05. Small and large dots present rank-transformed data and medians while the error bars represent interquartile ranges.

Next, we investigated the effect of early ART on inflammatory markers and a marker of neuronal damage. In our cross-sectional analysis (including baseline samples), participants from the early ART group had lower levels of interleukin (IL)-6 (Fig 5 and Table 1, p = 0.03) and tumor necrosis factor (TNF)-α (Fig 5 and Table 1, p = 0.02) in CSF compared to participants from the late ART group. ART groups did not differ for any of the other soluble inflammatory markers in CSF (sCD163 and MCP-1) or blood (sCD163, IL-6, TNF-α and MCP-1) or for neurofilament light (NFL) in CSF (p>0.1; Table 1).

We also used the time to ART as a continuous variable to evaluate its association with the levels of the four cytokines. We observed higher IL-6 levels among participants with the longest time to ART start from EDI, collapsed across blood and CSF (b = 0.19, p = 0.02, η^2^_p_ = 0.16). When evaluated separately, this association was significant in CSF (p = 0.02, η^2^_p_ = 0.16), but not in blood (p = 0.54, η^2^_p_ = 0.01). Again the five covariates were included in the model to control for their potential effects. The CD4/CD8 ratio was significantly negatively correlated with IL-6 levels (b = -0.37, p = 0.05, η^2^_p_ = 0.12), while the other four were not correlated (all p-values>0.1, all η^2^_p_<0.07). Regardless of the covariate included in the model, the association between time to ART and IL-6 remained consistently significant (p-values<0.05).

**Fig 6:**
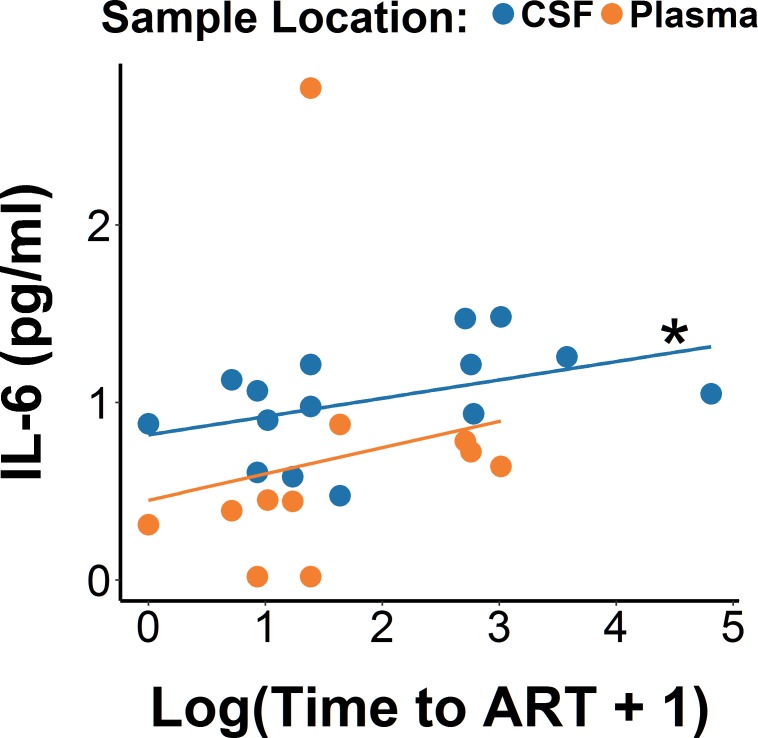
Regression analysis between time to ART start and IL-6 levels in blood and CSF. Linear mixed-effects regression models revealing relationship between levels of IL-6 with log-transformed time to ART start. *p<0.05 for the correlation within CSF.

Since IL-6 levels and HIV DNA diversity showed a similar, positive association with time to ART, we performed an additional mediation analysis to test the hypothesis that time to ART might have influenced diversity through its effect on IL-6 levels (Fig 6). While the direct effect of time to ART on diversity was still significant (p = 0.02), its indirect effect through IL-6 levels was not (p = 0.52), suggesting that IL-6 is unlikely the main mechanism connecting shorter timing of ART initiation to lover HIV DNA diversity.

## Discussion

To cure HIV, all forms of viral persistence should be considered, including viral reservoirs in different tissues and anatomical compartments [2,17,29–31]. Strong evidence supports that HIV can independently replicate in the CNS during untreated infection [2,11,32] and that the virus can establish a latent reservoir in this anatomic compartment [33,34], which may be distinct from the one in circulating CD4^+^ T cells. The exact timing of HIV compartmentalization within the CNS is uncertain but likely occurs soon after infection in at least some individuals [2,25]. Similarly to the periphery [35–38], we hypothesized that initiation of ART during early HIV infection would reduce the size and diversity of the viral reservoir within the CNS. To test this hypothesis, we evaluated a unique cohort of 16 HIV-infected individuals with known EDI who were sampled while receiving long-term ART and with sustained HIV RNA suppression. As previously described [39], we were able to detect HIV DNA in cells collected from the CSF, even in participants who started ART during early HIV infection (within 4 months of EDI). We did observe that early ART was associated with less molecular diversity of HIV DNA in both CSF cells and PBMC compared to late ART. Molecular diversity was not associated with age, peak viral load, CD4, CD8 and CD4/CD8 ratio.

Interestingly, although early ART initiation was associated with lower molecular diversity of provirus, most participants presented evidence of genetic compartmentalization of HIV DNA within the CSF (including 2 out of the 3 participants from the early ART group). Seven participants had a clear monophyletic population of HIV DNA in the CSF. Overall, our results are consistent with previous studies reporting the presence of compartmentalized HIV RNA in CSF of HIV-infected people very early after infection [2,25]. The detection of viral compartmentalization does not necessarily imply that the populations in CSF and in blood are completely segregated, but instead, distinct subpopulations can occur in each compartment. This can occur in two different ways. First, HIV RNA populations can be sequestered from blood and populate the CNS early after infection, giving rise to a HIV RNA population within the CSF that remains genetically distinct from blood throughout the course of infection [2]. Alternatively, HIV RNA can enter the CNS early and evolve over time as a consequence of isolated replication and differential selection pressures, creating a genetically complex population within the CNS [2]. Overall, these observations suggest that the CNS compartment is permissive for HIV replication in at least a subset of persons from a very early period after infection and likely originates a distinct reservoir from that found in the blood; however, it is noted in our study that we do not know if any of these HIV DNA sequences represented replication competent proviruses.

Another open question is the cellular source of this genetically distinct HIV DNA isolated from CSF cells. In our study, we were not able to determine the exact cellular source of the HIV DNA due to technical limitations and the nature of the samples. It is possible that this genetically distinct HIV DNA population detected in CSF might be carried by macrophages or T cells into the CSF or that T-cells circulating in CSF could get infected through contact with HIV-infected macrophages residing in the brain tissue in proximity to the brain vessels [40]. Alternatively, this HIV DNA population might be originating from CD4^+^ T cells circulating in the CSF after crossing the blood brain barrier but this seems less likely, since HIV-infected CD4^+^ T cells trafficking from the periphery into the CNS should present an equilibrated viral population in comparison to blood, especially in the setting of suppressive ART. Alternatively, unrecognized isolated HIV replication within the CNS during the period before our study visit might be responsible for our observations. Unfortunately, we did not collect longitudinal CSF samples in time points previous our baseline study visits, as part of the study design.

The novelty of our study derives from the fact that we evaluated the HIV DNA populations from cells circulating in CSF and we demonstrated the presence of compartmentalized monophyletic HIV DNA populations in CSF from HIV-infected participants receiving suppressive ART, including two participants who started ART during primary infection. Both participants with longitudinal sampling showed sustained compartmentalization at all time-points, and the same monophyletic population was repeatedly sampled from CSF over a period of 5 months in one participant.

Despite several technical limitations (described below), our findings are important for the design of future eradication strategies and also to improve our understanding of HIV pathogenesis in the CNS. In fact, the presence of compartmentalized HIV populations has been associated with neurocognitive impairment [15, 41]. Several studies reported associations between circulating HIV DNA levels in blood and neurocognitive impairment with and without ART [42–46]. While this observation might hold true also for HIV DNA in CNS, this has not been consistently reported especially in the setting of suppressive ART. One previous study [3], found higher levels of HIV DNA in brain tissue from people with HIV encephalitis and moderate neurocognitive impairment compared to HIV-positive controls dying without neurologic symptoms. However, this study was limited since it included autopsy material from people dying with advanced disease and variable ART exposure. Likely due to limitations in samples size and the fact that people treated early during HIV infection have overall less neurological complications, we did not find associations between HIV DNA levels and neurological impairment.

Our study also evaluated the effect of early ART initiation on selected inflammatory biomarkers in CSF and blood. Increased inflammation has been extensively reported in the CNS during HIV and was often associated with neurocognitive impairment [47–49] even during suppressive ART [49,50]. In our study, the early ART group presented significantly lower levels of IL-6 and TNF-α in CSF (but not in blood) compared to the late ART group. We also explored the possible effect of IL-6 on molecular diversity and no mediation effect was observed. These data further support the concept that early ART initiation reduces the levels of at least some inflammatory mediators in CSF.

This study has several limitations. First of all, even though we were able to collect the volumes of CSF necessary to recover sufficient cells by the lysis buffer protocol, the detection of HIV DNA from CSF has been challenging due the low number of cells typically present in CSF in the absence of neurological symptoms and when HIV is suppressed. The low number of input cells might increase the potential for error related to sampling bias, could possibly amplify the number of false positive events from the ddPCR assay and could affect our diversity and compartmentalization analysis. To partially evaluate its impact, we performed multiple sensitivity analysis to address a possible bias in our analysis. Although we acknowledge that the small samples size has limited our statistical evaluation, our primary predictor of interest (i.e. the time to ART initiation) appears to have a greater effect on molecular diversity than the assay-related covariates. Further, we significantly elevated the threshold of compartmentalization detection and specifically included computational tests to increase robustness against significant errors in frequency estimation. Template input was particularly low in some (but not all) CSF samples, which could negatively impact our capacity to find unique clades within the CSF: assuming we are simply resampling the most common variants, we are more likely to find that CSF sequences fall within better sampled blood variants. In contrast, despite the possible sampling bias in CSF, we were still able to observe monophyletic CSF variants at baseline in several participants. Also, the reproducibility of the phylogenetic trees with similar variants sampled across longitudinal CSF samples for one participant, suggests that our sequences are likely informative and not substantially affected by random error or sequencing bias. Despite this, and the fact that we are analyzing only a partial region of *env* gene (~400 bp), we found differences in molecular diversity of the HIV DNA populations in CSF between the early and late ART groups.

Another limitation of the analysis is the lack of randomization for the timing of ART initiation, which might introduce some unrecognized biases in our study design. For example, people with more symptomatic infection (including the presence of neurologic symptoms, which were not tested as part of our study) will start ART earlier and might also be more likely to present compartmentalized HIV populations. The small sample size also limited our statistical power. Even though some comparisons did not reach statistical significance, effect sizes were medium to large in some cases, supporting that the study was underpowered to answer these questions.

Another limitation is inherent in all CSF studies: CSF only approximates events in the brain. Despite this, CSF has provided many important insights into brain events in HIV and other diseases [51]. A high degree of HIV DNA compartmentalization within the CSF suggests that the sampled HIV DNA is originating from brain tissue, but it could also reflect a population of cells that preferentially migrate into CSF from blood. This will need to be evaluated in future studies using larger cohorts and post-mortem brain tissues. Finally, in this study, we were also not able to determine if the HIV DNA population sampled in the CSF is replication competent.

Despite these limitations, our data provide a unique perspective by analyzing HIV DNA populations sampled using CSF prospectively collected from a unique cohort of individuals who started ART and with known EDI. Our study supports the idea that initiation of ART during early infection may limit the diversity of HIV populations and inflammation in CNS. Future studies may want to evaluate the CSF HIV DNA populations in bigger cohorts and include longitudinal assessments prior and after initiation of ART to characterize dynamics of the CNS as a HIV reservoir. Moreover, future studies need to assess the CNS replication competent HIV DNA populations. The presence of unique HIV DNA populations within the CSF during ART might be relevant for future eradication strategies.

## Material and Methods

### Ethics statement

The study was approved by the Institutional Review Board at the University of California. All adult participants (age ≥ 18 years) provided written informed consent. No children were included in this study.

### Study cohort

Study participants were selected among HIV-infected men who enrolled in the SD PIRC between 2001 and 2012 and were still engaged in follow-up [52]. All SD PIRC participants are recruited during primary infection and followed with longitudinal blood drawn. Per protocol, visits occur at weeks 1, 2, 4, 8, 12, and 24, and then every 24 weeks thereafter. The date of infection is estimated for each participant following an established algorithm (summarized in supplementary S4 Table) [36]. Although early ART initiation is encouraged for all SD PIRC participants, implementation is based on participants’ personal decision, primary care physician input and following the current ART guidelines at the time of recruitment. Participants started ART between 2003 and 2012. Selection criteria for this study were: (1) HIV-infected males recruited during primary infection, (2) started ART during follow-up early or later during HIV-infection, (3) reached undetectable HIV RNA in blood plasma (<50 HIV RNA copies/ml) and remained undetectable during follow-up until the time of baseline CSF collection (based on our longitudinal viral loads and participant self-report) [53]. None of the participants had evidence of other inflammatory neurologic disorders or pleocytosis.

Participants were divided in early ART versus late ART groups as follow: 9 were included in the early ART group (≤4 months from estimated date of infection [EDI]) and 7 in late ART group (>14 months from EDI).

### Sample collection and processing

Paired blood and CSF samples were collected from each HIV-infected participant cross-sectionally. A subset of 2 participants provided a second pair of samples (3 and 5 months after their first evaluation, respectively) and one participant provided a third pair of samples (2 months thereafter).

We designed our study to maximize cellular recovery by collecting 40 ml of CSF fluid by lumbar puncture. Following standard procedures at the HIV Neurobehavioral Research Center (HNRC), the LPs were performed using atraumatic needle by an experienced physician. None of our study participants reported any complication following the CSF collection. From this larger volume, we obtained a CSF cell pellet and split it into two separate aliquots. Cell pellet lysates (containing HIV DNA) were used for ddPCR and for C2V3 *env* nested PCR as described below (see supplementary S1 Fig).

CSF supernatant was used to measure levels of selected markers of inflammation and neuronal damage (described below) and to measure HIV RNA by Aptima HIV RNA assay (Hologic), after concentrating 5 ml of supernatant (with single copy sensitivity). The CNS penetration effectiveness (CPE) index for the most recent ART regimen was determined as previously described [54]. For all participants, blood CD4+ T-lymphocytes were measured by flow-cytometry (CLIA certified local laboratory). Levels of HIV RNA in blood plasma were quantified by the Amplicor HIV Monitor Test (Roche Molecular Systems Inc.).

### Neurocognitive functioning

For each participant, neurocognitive functioning was assessed using a standardized clinical battery of seven ability areas consistent with Frascati recommendations for neuroAIDS research [55] and summarized using the validated global deficit score (GDS) [56].

### Levels of inflammation and neurofilament light chain

The levels of selected markers of monocyte activation (sCD163), general inflammation (IL-6) and (TNF-α) and monocyte trafficking monocyte chemoattractive protein (MCP)-1 as well as brain damage (NFL chains were measured in all participants. Enzyme-linked immunosorbent assay (ELISA) was used to quantify the levels of sCD163 (Trillium Diagnostics, Brewer, ME, USA) from blood plasma and CSF, and NFL in CSF (Uman Diagnnostics, Sweden). Electrochemiluminescence multiplex assay (Meso Scale Diagnostics, Rockville, MD, USA) was used to quantify the levels of IL-6, TNF-α and MCP-1 in CSF supernatant and blood plasma. All assessments were performed according to the manufacturer’s procedures.

### Quantification of HIV DNA from PBMC and CSF cellular pellets

Genomic DNA was extracted from 5 million PBMC for each participant (QIAmp DNA Mini Kit, Qiagen, CA) per manufacturer's protocol. Genomic DNA was also extracted from 1 (out of 2) aliquot of cell pellets obtained from 20 mL of CSF (in average, there were 34,000 white blood cells/aliquot, range: 20,000–60,000) using direct lysis as previously described [22, 23]. Levels of HIV DNA (*pol* gene region: HXB2 coordinates 2536–2662) were measured in triplicate by (dd)PCR [57]. Briefly, 5 μL of 1:2 diluted CSF lysates or 1000 ng of DNA from PBMC per replicate was digested with BANII enzyme (New England Biolabs) prior to ddPCR. Reactions were performed with the following cycling conditions: 10 minutes at 95°C, 40 cycles consisting of a 30 second denaturation at 94°C followed by a 60°C extension for 60 seconds, and a final 10 minutes at 98°C. For DNA from CSF cell pellets, we used 5 μL (diluted 1:2) of lysate per replicate. A 1:10 dilution of the digested DNA was used for host cell RPP30 (ribonuclease P30) ddPCR and cycled with same parameters described above. Copy numbers were calculated as the mean of the three PCR replicates measurements and normalized to one million of cells (PBMC or CSF cells) as determined by RPP30 levels. The limit of detection of the ddPCR assay for HIV DNA using the same primer-probe set was previously described as 0.7 copies per million of cells [57]. The detected number of RPP30 copies in each ddPCR reaction was used to estimate the number of cells per aliquot of CSF cellular pellet.

### Viral sequencing, sequence filtering and bioinformatics analysis

We amplified the HIV-1 *env* C2-V3 (HXB2 coordinates 6928–7344) region from DNA extracted from CSF cellular pellets and PBMC by nested PCR using specific primers [58]. Sequencing was performed using 454 GS FLX Titanium (454 Life Sciences, Roche, Branford, Connecticut, USA). Read (FASTA) and quality score files produced by the 454 instruments were further analyzed using a purpose-built bioinformatics pipeline [25–27]. The pipeline is available at *https://github.com/veg/HIV-NGS* and the key steps were summarized briefly bellow:

Raw data were filtered by removing sequences of low quality (q-score of less than 15) using the Datamonkey analysis tool [59] and aligned to a subtype B reference sequence [60]. High-quality reads were retained and aligned to HXB2 as a reference sequence (without generation of contigs) using an iterative codon-based alignment procedure implemented in Datamonkey. A Bayesian Dirichlet mixture of multinomials probabilistic model was used to distinguish sequencing error from true low-frequency variants (posterior probabilities of ≥99.99%). For PBMC, we obtained a median of reads of 16927.5 [13725, 23106.5] and for CSF, we obtained a median of reads of 16198 [9590, 20157.5]. All sets of representative reads were screened for evidence of recombination using GARD [29], APOBEC signatures, hypermutations and frame-shifts as part of our pipeline procedure. All sequences were screened for in-house cross-contamination using BLAST [61]. Identical sequence reads were clustered, allowing identification of non-redundant sequences. A minimum of 10 identical sequence reads were clustered into haplotypes, and the proportion of reads in each haplotype was provided. Hence, the output consists of a list of representative haplotypes and their relative frequencies. The average number of HIV DNA haplotypes recovered from the CSF is 21 (range: 11–29), while 27 (range: 9–46) haplotypes were recovered from blood. For each sample, we computed the mean of all pairwise Tamura-Nei 93 distances between reads with at least 100 overlapping base pairs to quantify nucleotide diversity [62].

### Compartmentalization analysis

Viral compartmentalization was first assessed by the Fst approach defined as $F_{ST}=1-\frac{\pi_{I}}{\pi_{D}}$, where π_I_ is the estimate of mean pairwise *intra-compartment* genetic distance (TN93) [28], and π_D_ is its *inter-compartment* counterpart [63]. Both quantities were computed by comparing all reads from blood and CSF compartments, subject to the requirement that they share at least 150 aligned nucleotide positions. The large number of pairwise comparisons (10^7^−10^9^) was handled computationally using an efficient implementation of the TN93 distance calculator (github.com/veg/tn93), which achieves a throughput of 10^7^ distances/second on a modern multi-core desktop. Subsequently, to guard against inference of compartmentalization by skewing of allelic frequencies due to PCR amplification and other biases, we recomputed F_ST_ by discarding copy number counts for read clusters (i.e. each cluster was counted as having only one sequence), i.e. all haplotypes are assigned a relative weight of 1. Statistical significance of both tests was derived via 1,000 population-structure randomization/permutation test. Finally, we performed a second tree-based Slatkin-Maddison (SM) test for compartmentalization [64]. Conservatively, we defined a CSF sample as compartmentalized only if all of the following tests were consistent and significant: (1) distance based F_ST_ test, (2) sensitivity test F_ST_ with collapsed haplotypes and (3) tree- based SM test.

### Phylogenetic analysis of partial *env* HIV-1 sequences

Viral haplotypes were realigned using MUSCLE [65], piped to FastTree 2 [66] for maximum likelihood trees reconstruction, and subjected to codon-based (MG94) phylogenetic analyses in HyPhy [67].

### Statistical analysis

Statistical differences between groups (early versus late ART initiation) were examined using linear mixed-effects models with individuals included as random intercepts. The time-to ART variable was dichotomized or log transformed, and outcome variables were rank-transformed when appropriate. When residual variance differed by a specific factor in analyzing untransformed outcomes, we allowed heterogeneous variances across levels of that factor. Differences for sparse variables were detected by Fisher exact test. Whenever possible, partial η^2^ (η^2^_p_) was provided as a measure of the strength of association. Statistical analyses were performed using the R statistical language ver 3.3 [68] and the nlme package [69].

## Supporting Information

S1 Fig CSF sample processing workflow. Forty milliliters of CSF were collected from HIV-infected people by lumbar puncture and CSF cells were pelleted down. CSF supernatant was stored at -80°C and subsequently used to measure levels of inflammation and neuronal damage as well as HIV RNA using a Single Copy Assay (Hologic). Cells were resuspended in freezing media and divided into two aliquots stored at -150°C. After thawing, cells were washed with PBS and centrifuged to obtain a dry CSF cell pellet. Cell pellets were directly lysed with lysis buffer and DNA was quantified by ddPCR and amplified by C2V3 Nested PCR.
(TIFF)

S1 Table Summary of positive samples by ddPCR and C2V3 *env* Nested PCR. (DOCX)

S2 Table Data summary for PBMC sequences. (DOCX)

S3 Table Data summary for CSF cellular pellets sequences. (DOCX)

S4 Table Brief Summary of the Algorithm for Computing the Estimated Date of HIV-1 Infection. (DOCX)

## References

[1] Spudich S, Gisslen M, Hagberg L, Lee E, Liegler T, Brew B, et al Central Nervous System Immune Activation Characterizes Primary Human Immunodeficiency Virus 1 Infection Even in Participants With Minimal Cerebrospinal Fluid Viral Burden. J Infect Dis. 2011;204: 753–760. 10.1093/infdis/jir387 21844301 PMC3156103

[2] Sturdevant CB, Joseph SB, Schnell G, Price RW, Swanstrom R, Spudich S. Compartmentalized Replication of R5 T Cell-Tropic HIV-1 in the Central Nervous System Early in the Course of Infection. PLoS Pathog. 2015;11: e1004720 10.1371/journal.ppat.1004720 25811757 PMC4374811

[3] Desplats P, Dumaop W, Smith D, Adame A, Everall I, Letendre S, et al Molecular and Pathologic Insights from Latent HIV-1 Infection in the Human Brain. Neurology. 2013;80: 1415–23. 10.1212/WNL.0b013e31828c2e9e 23486877 PMC3662272

[4] Bednar MM, Sturdevant CB, Tompkins LA, Arrildt KT, Dukhovlinova E, Kincer LP, et al Compartmentalization, Viral Evolution, and Viral Latency of HIV in the CNS. Curr HIV/AIDS Rep. 2015;12: 262–271. 10.1007/s11904-015-0265-9 25914150 PMC4431548

[5] Chamberland A, Sylla M, Boulassel MR, Baril J-G, Côté P, Thomas R, et al Effect of Antiretroviral Therapy on HIV-1 Genetic Evolution During Acute Anfection. International Journal of STD & AIDS. 2011 pp. 146–150. 21464451 10.1258/ijsa.2010.010292

[6] McNearney T, Hornickova Z, Markham R, Birdwell A, Arens M, Saah A, et al Relationship of Human Immunodeficiency Virus Type-1 Sequence Heterogeneity to Stage of Disease. ProcNatlAcadSci USA. 1992;89: 10247–10251. 10.1073/pnas.89.21.10247 PMC50315 1438212

[7] Schnell G, Spudich S, Harrington P, Price RW, Swanstrom R. Compartmentalized Human Immunodeficiency Virus Type 1 Originates from Long-Lived Cells in Some Subjects with HIV-1-Associated Dementia. PLoS Pathog. 2009;55(4): e1000395. 10.1371/journal.ppat.1000395 PMC2668697 19390619

[8] Sturdevant CB, Dow A, Jabara CB, Joseph SB, Schnell G, Takamune N, et al Central Nervous System Compartmentalization of HIV-1 Subtype C Variants Early and Late in Infection in Young Children. PLoS Pathog. 2012;8(12): e1003094 10.1371/journal.ppat.1003094 23300446 PMC3531524

[9] Harrington PR, Schnell G, Letendre SL, Ritola K, Robertson K, Hall C, et al Cross-sectional Characterization of HIV-1 Env Compartmentalization in Cerebrospinal Fluid Over the Full Disease Course. AIDS. 2009;23: 907–915. 10.1097/QAD.0b013e3283299129 19414991 PMC3089803

[10] Peters PJ, Sullivan WM, Duenas-Decamp MJ, Bhattacharya J, Ankghuambom C, Brown R, et al Non-Macrophage-Tropic Human Immunodeficiency Virus Type 1 R5 Envelopes Predominate in Blood, Lymph Nodes, and Semen: Implications for Transmission and Pathogenesis. J Virol. 2006;80: 6324–6332. 10.1128/JVI.02328-05 16775320 PMC1488974

[11] Smith DM, Zárate S, Shao H, Pillai SK, Letendre SL, Wong JK, et al Pleocytosis is Associated with Disruption of HIV Compartmentalization Between Blood and Cerebral Spinal Fluid Viral Populations. Virology. 2009;385: 204–208. 10.1016/j.virol.2008.11.010 19100592 PMC2794037

[12] Bull ME, Heath LM, McKernan-Mullin JL, Kraft KM, Acevedo L, Hitti JE, et al Human Immunodeficiency Viruses Appear Compartmentalized to the Female Genital Tract in Cross-Sectional Analyses but Genital Lineages Do Not Persist Over Time. J Infect Dis. 2013;207(8): 1206–1215. 10.1093/infdis/jit016 23315326 PMC3603533

[13] Heath L, Fox A, McClure J, Diem K, Wout AB, Zhao H, et al Evidence for Limited Genetic Compartmentalization of HIV-1 Between Lung and Blood. PLoS One. 2009;4: e6949 10.1371/journal.pone.0006949 19759830 PMC2736399

[14] Penton PK, Blackard JT. Analysis of HIV Quasispecies Suggests Compartmentalization in the Liver. AIDS Res Hum Retroviruses. 2014;30: 394–402. 10.1089/AID.2013.0146 24074301 PMC5972776

[15] Ritola K, Robertson K, Fiscus SA, Swanstrom R, Hall C. Increased Human Immunodeficiency Virus Type 1 (HIV-1) env Compartmentalization in the Presence of HIV-1-Associated Dementia. J Virol. 2005;1: 10830–10834.

[16] Strain MC, Letendre S, Pillai SK, Russell T, Ignacio CC, Gu HF, et al Genetic Composition of Human Immunodeficiency Virus Type 1 in Cerebrospinal Fluid and Blood Without Treatment and During Failing Antiretroviral Therapy. J Virol. 2005;79: 1772–1788. 10.1128/JVI.79.3.1772-1788.2005 15650202 PMC544082

[17] Schnell G, Joseph S, Spudich S, Price RW, Swanstrom R. HIV-1 Replication in the Central Nervous System Occurs in two Distinct Cell Types. PLoS Pathog. 2011;7: e1002286 10.1371/journal.ppat.1002286 22007152 PMC3188520

[18] Lanier ER, Sturge G, McClernon D, Brown S, Halman M, Sacktor N, et al HIV-1 Reverse Transcriptase Sequence in Plasma and Cerebrospinal Fluid of Patients with AIDS Dementia Complex Treated with Abacavir. AIDS. 2001;15(6): 747–51. 11371689 10.1097/00002030-200104130-00010

[19] Venturi G, Catucci M, Romano L, Corsi P, Leoncini F, Valensin P, et al Antiretroviral Resistance Mutations in Human Immunodeficiency Virus Yype 1 Reverse Transcriptase and Protease from Paired Cerebrospinal Fluid and Plasma Samples. J Infect Dis. 2000;181(2): 740–745. 10.1086/315249 10669367

[20] Di Stefano M, Sabri F, Leitner T, Svennerholm B, Hagberg L, Norkrans G, et al Reverse Transcriptase Sequence of Paired Isolates of Cerebrospinal Fluid and Blood from Patients Infected with Human Immunodeficiency Virus Type 1 During Zidovudine Treatment. J Clin Microbiol. 1995;33: 352–355. 7536214 10.1128/jcm.33.2.352-355.1995 PMC227947

[21] Sacktor N, McDermott MP, Marder K, Schifitto G, Selnes OA, McArthur JC, et al HIV-Associated Cognitive Impairment Before and After the Advent of Combination Therapy. J Neurovirol. 2002;8(2):136–42. 10.1080/13550280290049615 11935465

[22] Robertson KR, Smurzynski M, Parsons TD, Wu K, Bosch RJ, Wu J, et al The Prevalence and Incidence of Neurocognitive Impairment in the HAART Era. AIDS. 2007;21(14): 1915–21. 10.1097/QAD.0b013e32828e4e27 17721099

[23] Saylor D, Dickens AM, Sacktor N, Haughey N, Slusher B, Pletnikov M, et al HIV-associated neurocognitive disorder—pathogenesis and prospects for treatment. Nat Rev Neurol. 2016;12: 234–48. 10.1038/nrneurol.2016.27 26965674 PMC4937456

[24] Valcour V, Chalermchai T, Sailasuta N, Marovich M, Lerdlum S, Suttichom D, et al Central Nervous System Viral Invasion and Inflammation During Acute HIV Infection. J Infect Dis. 2012;206(2): 275–282. 10.1093/infdis/jis326 22551810 PMC3490695

[25] Schnell G, Price RW, Swanstrom R, Spudich S. Compartmentalization and Clonal Amplification of HIV-1 Variants in the Cerebrospinal Fluid During Primary Infection. J Virol. 2010;84(5): 2395–2407. 10.1128/JVI.01863-09 20015984 PMC2820937

[26] Jain V, Hartogensis W, Bacchetti P, Hunt PW, Hatano H, Sinclair E, et al Antiretroviral Therapy Initiated Within 6 Months of HIV Infection is Associated with Lower T-cell Activation and Smaller HIV Reservoir Size. J Infect Dis. 2013;208(8): 1202–1211. 10.1093/infdis/jit311 23852127 PMC3778965

[27] Hey-Cunningham WJ, Murray JM, Natarajan V, Amin J, Moore CL, Emery S, et al Early Antiretroviral Therapy with Raltegravir Generates Sustained Reductions in HIV Reservoirs but not Lower T-cell Activation Levels. AIDS. 2015;29(8): 911–9. 10.1097/QAD.0000000000000625 25730509

[28] Massanella M, Fromentin R, Chomont N. Residual Inflammation and Viral Reservoirs: Alliance Against an HIV Cure. Curr Opin HIV AIDS. 2016;11(2): 234–41. 10.1097/COH.0000000000000230 26575148 PMC4743501

[29] Carter CC, Mcnamara LA, Onafuwa-Nuga A, Iv JR, Bixby D, Savona MR, et al HIV-1 Utilizes the CXCR4 Chemokine Receptor to Infect Multipotent Hematopoietic Stem and Progenitor Cells. Cell Host Microbe. 2011;9(3): 223–234. 10.1016/j.chom.2011.02.005 21402361 PMC3102232

[30] Lerner P, Guadalupe M, Donovan R, Hung J, Flamm J, Prindiville T, et al The Gut Mucosal Viral Reservoir in HIV-Infected Patients is not the Major Source of Rebound Plasma Viremia Following Interruption of Highly Active Antiretroviral Therapy. J Virol. 2011;85(10): 4772–82. 10.1128/JVI.02409-10 21345945 PMC3126205

[31] Fomby P, Cherlin AJ. Hematopoietic Stem/Precursor cells as HIV Reservoirs. Curr Opin HIV AIDS. 2011;6(1): 43–48. 10.1097/COH.0b013e32834086b3 21242893 PMC3045752

[32] Schnell G, Price RW, Swanstrom R, Spudich S. Compartmentalization and Clonal Amplification of HIV-1 Variants in the Cerebrospinal Fluid During Primary Infection. J Virol. 2010;84(5): 2395–407. 10.1128/JVI.01863-09 20015984 PMC2820937

[33] Churchill MJ, Gorry PR, Cowley D, Lal L, Sonza S, Purcell DFJ, et al Use of Laser Capture Microdissection to Detect Integrated HIV-1 DNA in Macrophages and Astrocytes from Autopsy Brain Tissues. J Neurovirol. 2006;12(2): 146–52. 10.1080/13550280600748946 16798676

[34] Thompson KA, Cherry CL, Bell JE, McLean CA. Brain Cell Reservoirs of Latent Virus in Presymptomatic HIV-Infected Individuals. Am J Pathol. 2011;179(4): 1623–1629. 10.1016/j.ajpath.2011.06.039 21871429 PMC3181362

[35] Archin NM, Vaidya NK, Kuruc JD, Liberty AL, Wiegand A, Kearney MF, et al Immediate Antiviral Therapy Appears to Restrict Resting CD4+ Cell HIV-1 Infection without Accelerating the Decay of Latent Infection. Proc Natl Acad Sci. 2012;109(24): 9523–9528. 10.1073/pnas.1120248109 22645358 PMC3386138

[36] Le T, Wright EJ, Smith DM, He W, Catano G, Okulicz JF, et al Enhanced CD4+ T-cell Recovery with Earlier HIV-1 Antiretroviral Therapy. N Engl J Med. 2013;368(3): 218–30 10.1056/NEJMoa1110187 23323898 PMC3657555

[37] Ananworanich J, Schuetz A, Vandergeeten C, Sereti I, de Souza M, Rerknimitr R, et al Impact of Multi-Targeted Antiretroviral Treatment on Gut T Cell Depletion and HIV Reservoir Seeding During Acute HIV Infection. PLoS One. 2012;7(3): e33948 10.1371/journal.pone.0033948 22479485 PMC3316511

[38] Hocqueloux L, Avettand-Fenoel V, Jacquot S, Prazuck T, Legac E, Melard A, et al Long-term Antiretroviral Therapy Initiated During Primary HIV-1 Infection is Key to Achieving Both Low HIV Reservoirs and Normal T Cell Counts. J Antimicrob Chemother. 2013;68(5):1169–1178. 10.1093/jac/dks533 23335199

[39] De Oliveira MF, Gianella S, Letendre S, Scheffler K, Pond SLK, Smith DM, et al Comparative Analysis of Cell-Associated HIV DNA Levels in Cerebrospinal Fluid and Peripheral Blood by Droplet Digital PCR. PLoS One. 2015;10(10): 1–10. 10.1371/journal.pone.0139510 PMC4592012 26431315

[40] Kramer-Hämmerle S, Rothenaigner I, Wolff H, Bell JE, Brack-Werner R. Cells of the Central Nervous System as Targets and Reservoirs of the Human Immunodeficiency Virus. Virus Res. 2005;111: 194–213. 10.1016/j.virusres.2005.04.009 15885841

[41] Pillai SK, Pond SLK, Liu Y, Good BM, Strain MC, Ellis RJ, et al Genetic Attributes of Cerebrospinal Fluid-Derived HIV-1 env. Brain. 2006;129: 1872–83. 10.1093/brain/awl136 16735456

[42] Valcour VG, Ananworanich J, Agsalda M, Sailasuta N, Chalermchai T, Schuetz A, et al HIV DNA reservoir increases risk for cognitive disorders in cART-naïve patients. PLoS One. 2013;8: e70164 10.1371/journal.pone.0070164 23936155 PMC3729685

[43] Valcour VG, Shiramizu BT, Shikuma CM. HIV DNA in circulating monocytes as a mechanism to dementia and other HIV complications. J Leukoc Biol. 2010;87(4): 621–626. 10.1189/jlb.0809571 20130221 PMC2858306

[44] Shiramizu B, Paul R, Williams A, Shikuma C, Watters M, Grove J, Valcour V. HIV Proviral DNA Associated With Decreased Neuropsychological Function. J Neuropsychiatry Clin Neurosci. 2007;19(2): 157–163. 10.1176/appi.neuropsych.19.2.157 17431062 PMC2399894

[45] Shiramizu B, Williams AE, Shikuma C, Valcour V. Amount of HIV DNA in Peripheral Blood is Proportional to the Severity of Neurocognitive Disorders. J Neuropsychiatry Clin Neurosci. 2009;21(1): 68–74. 10.1176/appi.neuropsych.21.1.68 19359454 PMC2668129

[46] Oliveira MF, Murrel B, Pérez-Santiago J, Vargas M, Ellis RJ, Letendre S, et al Circulating HIV DNA Correlates With Neurocognitive Impairment in Older HIV-infected Adults on Suppressive ART. Sci Rep. 2015;5: 17094 10.1038/srep17094 26603568 PMC4658529

[47] Cinque P, Vago L, Mengozzi M, Torri V, Ceresa D, Vicenzi E, et al Elevated Cerebrospinal Fluid Levels of Monocyte Chemotactic Protein-1 Correlate with HIV-1 Encephalitis and Local Viral Replication. AIDS. 1998;12: 1327–1332. 9708412 10.1097/00002030-199811000-00014

[48] Mehla R, Mehla SB, Nagarkatti M, Chauhan A. Programming of Neurotoxic Cofactor CXCL-10 in HIV-1-Associated Dementia: Abrogation of CXCL-10-Induced Neuro-Glial Toxicity in vitro by PKC Activator. J Neuroinflammation. 2012;9(1): 239. 23078780 10.1186/1742-2094-9-239 PMC3533742

[49] Burdo TH, Weiffenbach A, Woods SP LS, Ellis RJ and Williams KC. Elevated sCD163 in Plasma but not Cerebrospinal Fluid is a Marker of Neurocognitive Impairment in HIV Infection. AIDS. 2013;27(9): 1–16. 23435298 10.1097/QAD.0b013e32836010bd PMC3844286

[50] Gelman BB, Lisinicchia JG, Morgello S, Masliah E, Commins D, Achim CL, et al Neurovirological Correlation with HIV-Associated Neurocognitive Disorders and Encephalitis in a HAART-era Cohort. J Acquir Immune Defic Syndr. 2013;62(5): 487–95. 10.1097/QAI.0b013e31827f1bdb 23242157 PMC3664102

[51] Price RW, Staprans S. Measuring the “viral load” in Cerebrospinal Fluid in Human Immunodeficiency Virus Infection: Window into Brain Infection? Ann Neurol. 1997;42(5): 675–8. 10.1002/ana.410420502 9392565

[52] Morris SR, Little SJ, Cunningham T, Garfein RS, Richman DD, Smith DM. Evaluation of an HIV Nucleic Acid Testing Program with Automated Internet and Voicemail Systems to Deliver Results. Ann Intern Med. 2010;152(12): 778–785. 10.7326/0003-4819-152-12-201006150-00005 20547906 PMC2922925

[53] Little SJ, Frost SDW, Wong JK, Smith DM, Pond SLK, Ignacio CC, et al Persistence of Transmitted Drug Resistance among Subjects with Primary Human Immunodeficiency Virus Infection. J. Virol. 2008;82(11): 5510–5518. 10.1128/JVI.02579-07 18353964 PMC2395184

[54] Letendre S, Marquie-Beck J, Capparelli E, Best B, Clifford D, Collier AC, et al Validation of the CNS Penetration-Effectiveness Rank for Quantifying Antiretroviral Penetration Into the Central Nervous System. Arch Neurol. 2009;65(1): 65–70. 10.1001/archneurol.2007.31 PMC2763187 18195140

[55] Antinori A, Arendt G, Becker JT, Brew BJ, Byrd DA, Cherner M, et al Updated Research Nosology for HIV-Associated Neurocognitive Disorders. Neurology. 2007;69(18): 1789–99. 10.1212/01.WNL.0000287431.88658.8b 17914061 PMC4472366

[56] Blackstone K, Moore DJ, Franklin DR Jr, Clifford DB, Collier AC, Marra CM, et al Defining Neurocognitive Impairment in HIV: Deficit Scores versus Clinical Ratings. Clin Neuropsycology. 2012;26(6): 1–17. 10.1080/13854046.2012.694479 PMC3848322 22708483

[57] Strain MC, Lada SM, Luong T, Rought SE, Gianella S, Terry VH, et al Highly Precise Measurement of HIV DNA by Droplet Digital PCR. PLoS One. 2013;8(4): e55943 10.1371/journal.pone.0055943 23573183 PMC3616050

[58] Gianella S, Delport W, Pacold ME, Young JA, Choi JY, Little SJ, et al Detection of Minority Resistance During Early HIV-1 Infection: Natural Variation and Spurious Detection Rather than Transmission and Evolution of Multiple Viral Variants. J Virol. 2011;85(16): 8359–8367. 10.1128/JVI.02582-10 21632754 PMC3147985

[59] Delport W, Poon AFY, Frost SDW, Kosakovsky Pond SL. Datamonkey 2010: A Suite of Phylogenetic Analysis Tools for Evolutionary Biology. Bioinformatics. 2010;26(19): 2455–2457. 10.1093/bioinformatics/btq429 20671151 PMC2944195

[60] Fisher RG, Smith DM, Murrell B, Slabbert R, Kirby BM, Edson C, et al Next Generation Sequencing Improves dDtection of Drug Resistance Mutations in Infants after PMTCT Failure. J Clin Virol. 2015;62: 48–53. 10.1016/j.jcv.2014.11.014 25542470 PMC4279108

[61] Smith D, Delport W, Butler D, Little S, Richman D, Pond SK. Response to Comment on “The Origins of Sexually Transmitted HIV Among Men Who Have Sex with Men.” Sci Transl Med. 2010;2(50): 501r1 10.1126/scitranslmed.3001473 21532938 PMC3083242

[62] Tamura K, Nei M. Estimation of the Number of Nucleotide Substitutions in the Control Region of Mitochondrial DNA in Humans and Chimpanzees. Mol Biol Evol. 1993;10(3): 512–526. 8336541 10.1093/oxfordjournals.molbev.a040023

[63] Hudson RR. A New Statistic for Detecting Genetic Differentiation. Genetics. 2000;155(4): 2011–2014. 10924493 10.1093/genetics/155.4.2011 PMC1461195

[64] Slatkin M. Isolation by Distance in Equilibrium and Non-Equilibrium Populations. Evolution. 1993;47(1): 264–279. 28568097 10.1111/j.1558-5646.1993.tb01215.x

[65] Edgar RC. MUSCLE: A Multiple Sequence Alignment Method with Reduced Time and Space Complexity. BMC Bioinformatics. 2004;5:113. 15318951 10.1186/1471-2105-5-113 PMC517706

[66] Guindon S, Gascuel O. A Simple, Fast, and Accurate Algorithm to Estimate Large Phylogenies by Maximum Likelihood. Syst Biol. 2003;52(5):696–704. 14530136 10.1080/10635150390235520

[67] Pond SL, Frost SD, Muse SV. HyPhy: Hypothesis Testing Using Phylogenies. Bioinformatics. 2005;21(5):676–9. 10.1093/bioinformatics/bti079 15509596

[68] https-:www.R-project.org:.pdf.

[69] http://CRAN.R-project.org:package=nlme.pdf.

