# Supplementary figures and images for: Early Antiretroviral Therapy Is Associated with Lower HIV DNA Molecular Diversity and Lower Inflammation in Cerebrospinal Fluid but Does Not Prevent the Establishment of Compartmentalized HIV DNA Populations
Source: PLoS Pathog. 2017 Jan 3;13(1):e1006112. doi: 10.1371/journal.ppat.1006112 (PMC5266327; doi:10.1371/journal.ppat.1006112)

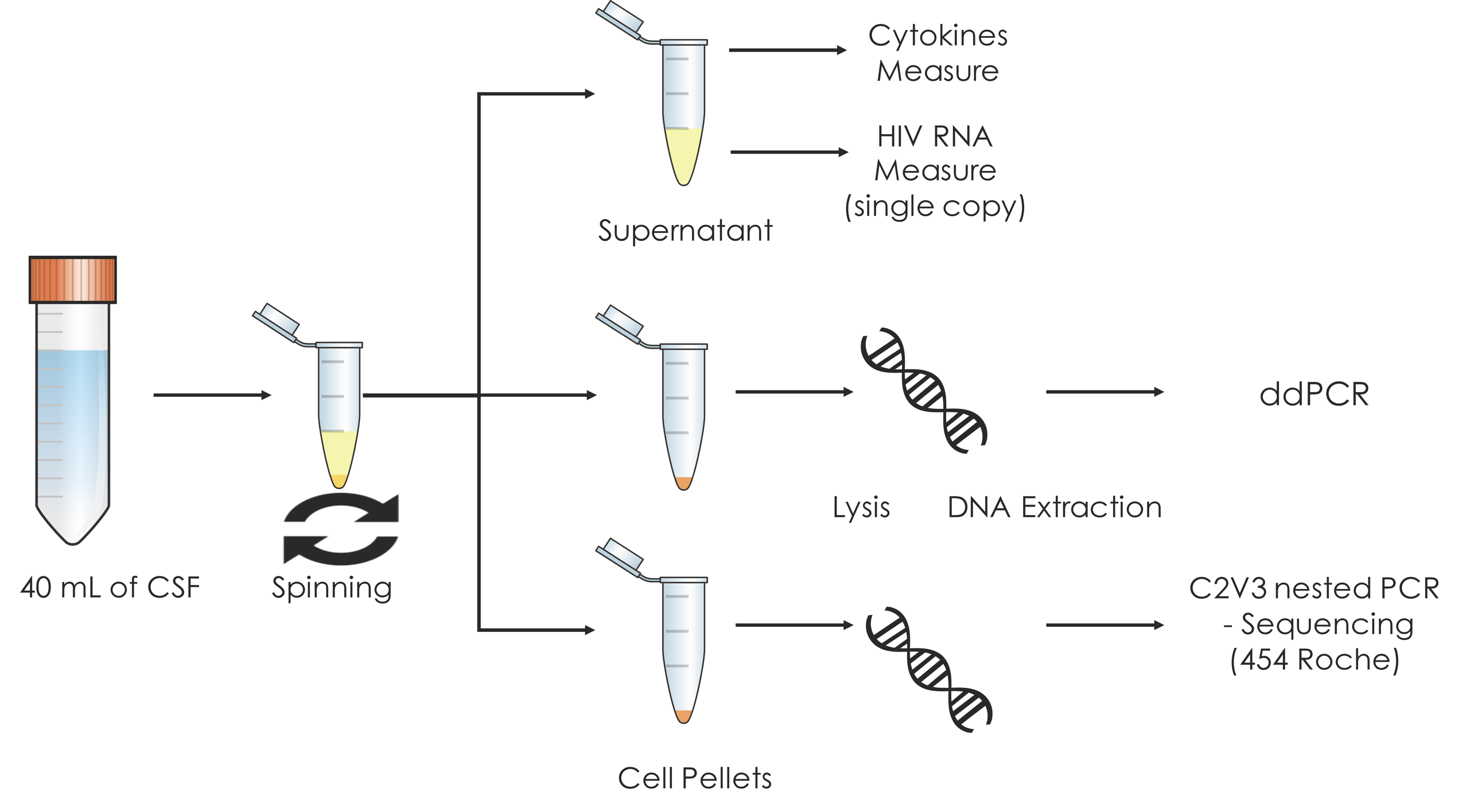

Supplement: S1 Fig — Forty milliliters of CSF were collected from HIV-infected people by lumbar puncture and CSF cells were pelleted down. CSF supernatant was stored at -80°C and subsequently used to measure levels of inflammation and neuronal damage as well as HIV RNA using a Single Copy Assay (Hologic). Cells were resuspended in freezing media and divided into two aliquots stored at -150°C. After thawing, cells were washed with PBS and centrifuged to obtain a dry CSF cell pellet. Cell pellets were directly lysed with lysis buffer and DNA was quantified by ddPCR and amplified by C2V3 Nested PCR. (TIFF) [file ppat.1006112.s001.tiff]
