# Supplementary material for: Early Antiretroviral Therapy Is Associated with Lower HIV DNA Molecular Diversity and Lower Inflammation in Cerebrospinal Fluid but Does Not Prevent the Establishment of Compartmentalized HIV DNA Populations
Source: PLoS Pathog. 2017 Jan 3;13(1):e1006112. doi: 10.1371/journal.ppat.1006112 (PMC5266327; doi:10.1371/journal.ppat.1006112)
Supplement: S1 Table — (DOCX) [file ppat.1006112.s002.docx]

| **Table S1. Summary of positive samples by ddPCR and C2V3 *env* Nested PCR.** | | | | | |
| --- | --- | --- | --- | --- | --- |
| **Subject** | **TP** | **Group** | **ddPCR** | **Nested PCR**  **C2V3** | **Detectable HIV DNA**  **(ddPCR and/or Nested PCR)** |
| T0430 | Baseline | Early | - | + | Yes |
| T0390 | Baseline | Early | - | - | - |
| T0190 | Baseline | Early | + | - | Yes |
| T0248 | Baseline | Early | - | - | - |
| T0073 | Baseline | Early | + | + | Yes |
| T0259 | Baseline | Early | - | - | - |
| T0417 | Baseline | Early | - | + | Yes |
| T0104 | Baseline | Early | - | + | Yes |
| T0349 | Baseline | Early | - | - | - |
| T0144 | Baseline | Late | - | - | - |
| T0238 | Baseline | Late | - | - | - |
| T0020 | Baseline | Late | - | + | Yes |
| T0133 | Baseline | Late | + | + | Yes |
| T0156 | Baseline | Late | + | + | Yes |
| T0338 | Baseline | Late | + | + | Yes |
|  | TP2 |  | + | + | Yes |
| T0366 | Baseline | Late | + | - | Yes |
|  | TP2 |  | - | + | Yes |
|  | TP3 |  | - | + | Yes |
