# Supplementary material for: Early Antiretroviral Therapy Is Associated with Lower HIV DNA Molecular Diversity and Lower Inflammation in Cerebrospinal Fluid but Does Not Prevent the Establishment of Compartmentalized HIV DNA Populations
Source: PLoS Pathog. 2017 Jan 3;13(1):e1006112. doi: 10.1371/journal.ppat.1006112 (PMC5266327; doi:10.1371/journal.ppat.1006112)
Supplement: S2 Table — (DOCX) [file ppat.1006112.s003.docx]

| **Table S2. Data summary for PBMC sequences** | | | | | | | | |
| --- | --- | --- | --- | --- | --- | --- | --- | --- |
|  |  |  | Diversity (%) | | | |  |  |
| **PID** | **Comp** | **TP** | **Overall** | **Syn** | **Non-Syn** | **TN93** |  | **Mean length (aa)** |
| T0020 | PBMC | 1 | 1.29 | 0.27 | 1.01 | 0.74 |  | 131.64 |
| T0073 | PBMC | 1 | 1.16 | 0.24 | 0.92 | 0.07 |  | 131.56 |
| T0104 | PBMC | 1 | 3.02 | 0.71 | 2.31 | 0.41 |  | 130.02 |
| T0133 | PBMC | 1 | 2.52 | 0.51 | 2.01 | 2.05 |  | 131.77 |
| T0144 | PBMC | 1 | 1.27 | 0.48 | 0.79 | 0.79 |  | 133.36 |
| T0156 | PBMC | 1 | 4.54 | 1.23 | 3.31 | 7.35 |  | 133.17 |
| T0190 | PBMC | 1 | 0.61 | 0.17 | 0.43 | 0.17 |  | 131.08 |
| T0238 | PBMC | 1 | 3.97 | 1.09 | 2.88 | 4.26 |  | 129.01 |
| T0248 | PBMC | 1 | 2.18 | 0.52 | 1.66 | 1 |  | 132 |
| T0259 | PBMC | 1 | 2.46 | 0.88 | 1.58 | 0.77 |  | 128.04 |
| T0338 | PBMC | 1 | 2.45 | 0.09 | 2.36 | 0.99 |  | 131.41 |
| T0338 | PBMC | 2 | 2.62 | 0.27 | 2.35 | 0.15 |  | 133.21 |
| T0349 | PBMC | 1 | 3.65 | 0.91 | 2.74 | 1.54 |  | 131.48 |
| T0366 | PBMC | 2 | 2.67 | 0.57 | 2.1 | 0.95 |  | 131.5 |
| T0366 | PBMC | 3 | 3.53 | 0.98 | 2.55 | 1.59 |  | 132.14 |
| T0430 | PBMC | 1 | 0.84 | 0.46 | 0.38 | 0.74 |  | 132.17 |
