# Supplementary material for: Early Antiretroviral Therapy Is Associated with Lower HIV DNA Molecular Diversity and Lower Inflammation in Cerebrospinal Fluid but Does Not Prevent the Establishment of Compartmentalized HIV DNA Populations
Source: PLoS Pathog. 2017 Jan 3;13(1):e1006112. doi: 10.1371/journal.ppat.1006112 (PMC5266327; doi:10.1371/journal.ppat.1006112)
Supplement: S3 Table — (DOCX) [file ppat.1006112.s004.docx]

| **Table S3. Data summary for CSF cellular pellets sequences** | | | | | | | | |
| --- | --- | --- | --- | --- | --- | --- | --- | --- |
|  |  |  | Diversity (%) | | | |  |  |
| **PID** | **Comp** | **TP** | **Overall** | **Syn** | **Non-Syn** | **TN93** |  | **Mean length (aa)** |
| T0020 | CSF | 1 | 1.09 | 0.47 | 0.62 | 1.03 |  | 131.9 |
| T0073 | CSF | 1 | 0.8 | 0.47 | 0.33 | 0.19 |  | 130.48 |
| T0104 | CSF | 1 | 0.94 | 0.23 | 0.71 | 0.06 |  | 131.02 |
| T0133 | CSF | 1 | 0.84 | 0.15 | 0.69 | 0.56 |  | 130.41 |
| T0156 | CSF | 1 | 3.83 | 1.35 | 2.48 | 3.05 |  | 132.4 |
| T0338 | CSF | 1 | 2.6 | 0.7 | 1.9 | 0.93 |  | 131.51 |
| T0338 | CSF | 2 | 2.05 | 0.26 | 1.79 | 1.11 |  | 131.96 |
| T0366 | CSF | 2 | 2.53 | 0.8 | 1.72 | 0.92 |  | 133.01 |
| T0366 | CSF | 3 | 0.56 | 0.23 | 0.33 | 0.52 |  | 132.7 |
| T0417 | CSF | 1 | 1.63 | 0.4 | 1.23 | 1.09 |  | 130.59 |
| T0430 | CSF | 1 | 0.49 | 0.38 | 0.11 | 0.41 |  | 132.09 |
