# Supplementary material for: Early Antiretroviral Therapy Is Associated with Lower HIV DNA Molecular Diversity and Lower Inflammation in Cerebrospinal Fluid but Does Not Prevent the Establishment of Compartmentalized HIV DNA Populations
Source: PLoS Pathog. 2017 Jan 3;13(1):e1006112. doi: 10.1371/journal.ppat.1006112 (PMC5266327; doi:10.1371/journal.ppat.1006112)
Supplement: S4 Table — (DOCX) [file ppat.1006112.s005.docx]

| **Table S4.** Brief Summary of the Algorithm for Computing the Estimated Date of HIV-1 Infection^a^ | |
| --- | --- |
| Class | Definitions for stage of HIV-1 infection |
| A1.0 | If there is a first positive RNA† and negative enzyme immunoassay (EIA) within 7 days of the first positive RNA, and no prior positive/indeterminate western blot (WB), then EDI = first positive RNA date − 11 days. (~Fiebig Stages I-II‡) |
| A2.0 | If there is an indeterminate WB within 7 days of the first positive RNA, then EDI = first positive RNA date − 20 days. (~Fiebig Stages III-IV‡) |
| A3.0 | If the last negative EIA or negative/indeterminate WB occurred ≤ 30 days before the first positive WB (with associated positive RNA), then EDI = midpoint of the positive WB date and the negative EIA or negative/indeterminate WB date (earlier of two) − 19 days. (~Fiebig Stage IV‡) |
| A3.1 | If the first positive WB p31/32 band is absent, then EDI = first positive WB date − 89 days. (~Fiebig Stage V‡) |
| E1.0A | If there is a detuned EIA (dtEIA) consistent with infection of ~3 mo within 30 days of the first positive WB and CD4 count > 200 or CD4% > 14 within 30 days of the first positive WB, then EDI = first dtEIA date − 70 days§. (Fiebig VI‡) |
| E1.0B | If there is a dtEIA consistent with infection of ~3-6 mo within 30 days of the first positive WB and CD4 count > 200 or CD4% > 14), then EDI = first dtEIA date − 133 days§. (Fiebig VI‡) |
| E1.0C | If there is a dtEIA consistent with infection of ~6-12 mo within 30 days of the first positive WB and CD4 count > 200 or CD4% > 14, then EDI = first dtEIA date − 170 days§. (Fiebig VI‡) |
| E2.0 | If there is a first positive WB and a negative EIA within 365 days participant enrollment (Day 0), then EDI = midpoint between the last negative EIA and Day 0. (Fiebig VI‡) |
| Each rule applied sequentially until EDI criteria satisfied.  † Positive RNA was defined as a NAT/viral load exceeding the detectable level for a given assay.  ‡ Indexed to algorithm from Fiebig et al. (70) Note: no endpoint was defined for stage VI by Fiebig.  § dtEIA threshold from Kothe et al. (71) | |
|  |  |

70. Fiebig EW, Wright DJ, Rawal BD, et al. Dynamics of HIV Viremia and Antibody Seroconversion in Plasma Donors: Implications for Diagnosis and Staging of Primary HIV Infection. AIDS. 2003; 17:1871–1879.

71. Kothe D, Byers RH, Caudill SP, et al. Performance Characteristics of a New Less Sensitive HIV-1 Enzyme Immunoassay for Use in Estimating HIV Seroincidence. J Acquir Immune Defic Syndr. 2003; 33:625–634.
